# Comparison of health behaviours between cancer survivors and the general population: a cross-sectional analysis of the Lifelines cohort

**DOI:** 10.1007/s11764-020-00854-2

**Published:** 2020-01-14

**Authors:** Francisco O. Cortés-Ibáñez, Daniel A. Jaramillo-Calle, Petra C. Vinke, Oyuntugs Byambasukh, Eva Corpeleijn, Anna Sijtsma, Christine Eulenburg, Judith M. Vonk, Geertruida H. de Bock

**Affiliations:** 1 Department of Epidemiology, University Medical Center Groningen, University of Groningen, Hanzeplein 1 (9713 GZ), Groningen, The Netherlands; 2 National Autonomous University of Mexico (UNAM), Mexico City, Mexico; 3 IPS Universitaria, University of Antioquia, Medellin, Colombia; 4 The Lifelines Cohort, University Medical Center in Groningen, Groningen, The Netherlands

**Keywords:** Neoplasms, Prevalence, Lifestyle, Health behaviour, Risk factors, Survivors

## Abstract

**Purpose:**

To compare the differences in lifestyle behaviours between cancer survivors (CSs) and cancer-free participants in a large and representative population-based cohort.

**Methods:**

We included 115,257 adults from the Lifelines cohort. Cancer status was self-reported, and health behaviours were measured (e.g. body mass index [BMI]) or assessed by questionnaire (e.g. physical activity, smoking, alcohol consumption, sedentary behaviour and diet). The data were then categorised for logistic regression analysis, stratified and adjusted by sex and age (< 55 vs ≥ 55 years).

**Results:**

CSs (5473; 4.7%) were diagnosed 9 ± 8.5 years before data collection, were older (mean age 55.4 vs 44.4 years) and more often female (66.6% vs 33.4%) than the cancer-free participants. They were also more likely to be physically active and to have a better diet, and also less likely to be alcohol drinkers; but, were more likely to have a higher BMI, be former smokers and to be sedentary. After adjustment for sex and age, however, BMI was more likely to be normal, physical activity was more likely to be higher and smoking to be prevalent in CSs. Current smoking was also significantly higher among females and those aged < 55 years who were CSs than for those with no history of cancer.

**Conclusions:**

In this population-based cohort, CSs have health behaviour comparable to those without a cancer diagnosis.

**Implications for cancer survivors:**

Smoking cessation strategies should target all CSs, but efforts could yield greatest benefit if they target females and those younger than 55 years.

## Introduction

The incidence of cancer is rising, due in large part to an ageing population [1]. Moreover, these patients tend to be surviving for longer; thanks to earlier detection, better diagnostic and staging methods and improved treatments [2]. About two-thirds of individuals will now survive longer than 5 years after a cancer diagnosis. Consequently, the number of cancer survivors (CSs) has increased over the past decade [3], with 771,046 recorded in the Netherlands [4] and 12.5 million recorded in all European countries [5] as of 2017.

There is extensive evidence that unhealthy lifestyle behaviours increase the risk of developing a primary cancer [6–10]. Such behaviours include alcohol intake [11], physical inactivity [12], sedentary behaviour [13, 14], unhealthy dietary patterns [15, 16], tobacco smoking [17] and being overweight or obese [18, 19]. An unhealthy lifestyle may not only contribute to the development of cancer but also to its recurrence in CSs, while simultaneously increasing the risk of cardiovascular disease, type 2 diabetes and other chronic diseases [20]. As such, it is essential that we better understand the prevalence and patterns of unhealthy lifestyle behaviour and promote a healthy lifestyle among CSs.

Many small-scale studies, but only a few population-based studies, have evaluated lifestyle behaviour among survivors of specific cancers, with a focus on breast cancer [21–23], colorectal cancer [23–25] and prostate cancer [23]. An increasing number of population-based studies have also evaluated lifestyle behaviours among CSs [26–33], and these have shown different results regarding the healthiness of the lifestyles of CSs compared to cancer-free groups. Whereas some studies have found that healthier behaviours predominate among CSs compared with cancer-free groups, with less alcohol drinking [30, 33], less smoking [26, 27, 29, 30, 33], better diets [31] and lower levels of inactivity [33], other studies have reported more unhealthy behaviours among CSs, such as being less physically active [27, 29, 31] or more previous alcohol drinking [26]. These contrasting results may be attributable to differences in sample sizes, cultures and sociodemographic characteristics among the populations. Moreover, although all studies included smoking, physical activity and alcohol intake [26–33], only four considered dietary intake [27–29, 31]; only one comprehensively evaluated diet [29], and three did not include body mass index (BMI) in their analysis [29, 30, 33]. Further research is therefore needed to overcome the limitations of existing studies in this field.

In the present analysis, we aimed to compare the differences in key lifestyle behaviours between a CSs group and a cancer-free group in a large and representative population-based cohort. We were particularly interested in six lifestyle factors: BMI, physical activity, smoking, alcohol consumption, sedentary behaviour and diet.

## Methods

### Study design

We conducted a cross-sectional analysis of data from Lifelines, a large prospective population-based cohort that was set up to examine the health and health-related behaviours of 10% of the population living in the north of the Netherlands [34, 35]. Using a unique three-generation design (i.e. grandparents, parents and children), this cohort employed a broad range of investigative procedures to allow future researchers assess the biomedical, sociodemographic, behavioural, physical and psychological factors that contribute to health and disease in the general population. However, there is a special focus on multimorbidity and complex genetics. The cohort protocol followed the principles of the Declaration of Helsinki and was approved by the medical ethics review committee of the University Medical Center Groningen.

### Participants

**Fig. 1:**
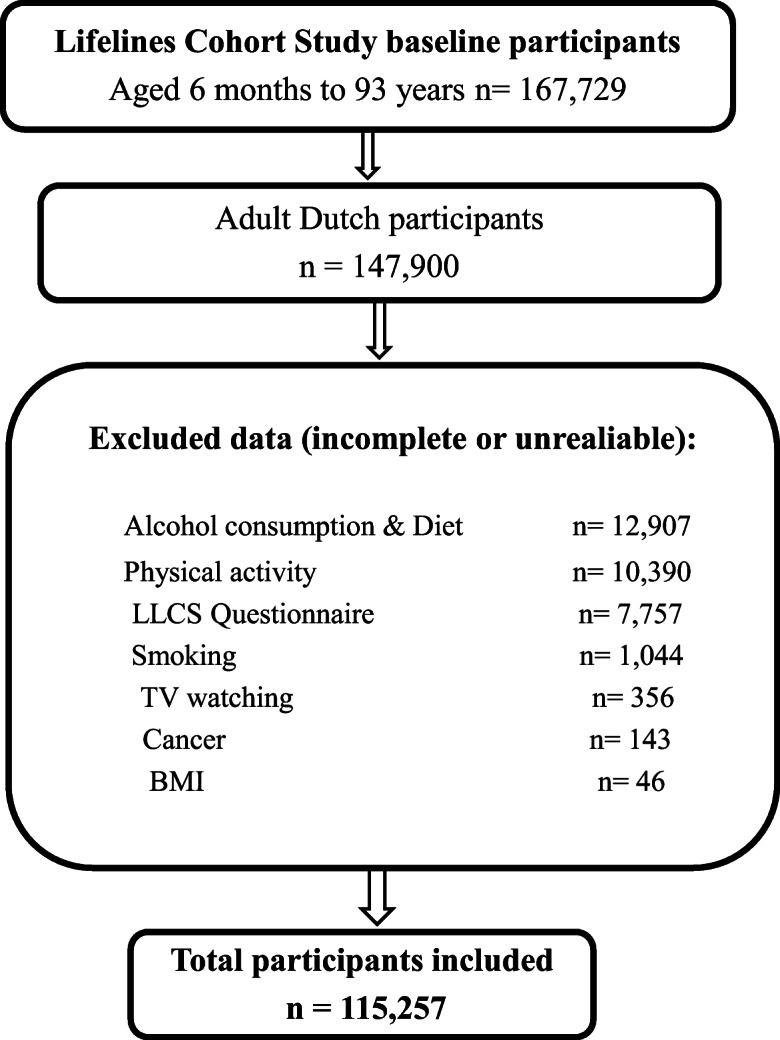
Participants selection

The Lifelines database includes data for 167,729 participants aged 6 months to 93 years collected between 2006 and 2013. We included the baseline data from adult participants (age ≥ 18 years, *n* = 147,900) with complete information for personal cancer history (yes/no) and the investigated lifestyle factors (*n* = 115,257 participants) (Fig. 1). Those participants who answered affirmatively when asked whether they had ever been diagnosed with cancer, who provided the time since cancer diagnosis and/or who stated they had been cured of cancer were considered as CSs.

### Data collection and parameters

All participants answered a self-administered two-part questionnaire to provide demographic, health status, lifestyle and psychosocial information. Height and body weight were measured at one of the Lifelines research sites. We then considered the following data in the present analysis: age, sex, BMI, smoking, alcohol intake, physical activity, sedentary behaviour, diet quality and personal cancer history. BMI was calculated by dividing weight by the height squared (BMI, kg/m^2^), and a BMI ≥ 25 kg/m^2^ was considered overweight.

Physical activity was evaluated by the Short Questionnaire to Assess Health-enhancing Physical Activity (SQUASH) [36], which has been validated in the Dutch population. This questionnaire includes items on routine activities such as commuting, leisure, household, work and school. According to the Dutch Health Council physical activity guideline, it is recommended that a person spends at least 150 min engaged in moderate-to-vigorous physical activity per week. We only considered moderate-to-vigorous activities related to commuting and leisure time because these domains better represent lifestyle behaviour than occupational physical activity [37]. Moderate-to-vigorous physical activity levels of < 150 and ≥ 150 min/week were considered low and high levels, respectively.

Smoking history was categorised into never, former and current. Never smokers were defined as those who answered ‘no’ to the question ‘Have you ever smoked for as long as a year?’. Former smokers were defined as those who reported being smokers for more than a year but had stopped smoking for at least 1 month at the time of the questionnaire. Current smokers were defined as those who answered ‘yes’ to the question ‘Do you smoke now or have you smoked in the last month?’ [38].

Alcohol intake was measured in grams of alcohol consumed per day and was evaluated based on the Lifelines 110-item food-frequency questionnaire (FFQ), which assessed food intake over the previous month. Participants who reported drinking < 1 g/day in the previous month were considered non-consumers and those who reported ≥ 1 g/day in the previous month were considered consumers. In this question, 1 g of alcohol per day was based on three standard drinks per month where a standard unit of alcohol was 10 g per drink in the Netherlands [39].

Sedentary behaviour was evaluated by the number of hours watching TV per day, which has been related to cancer risk independently of leisure time physical activity [14]. Watching TV for ≥2 h/day was considered to indicate sedentary behaviour.

Diet quality was assessed by the food-based Lifelines Diet Score (LLDS), which was calculated with the FFQ data. The LLDS rates the relative intakes of different food groups with known health effects—either positive (e.g. vegetables, fruit, whole grain products, legumes and nuts, fish, oils and soft margarines, unsweetened dairy, coffee and tea) or negative (e.g. red and processed meat, butter, hard margarines and sugar-sweetened beverages) on a scale ranging from 0 (lowest diet quality) to 48 (highest diet quality) [40]. A score below the sample mean was considered to indicate a low-quality diet, whereas a score equal to or higher than the sample mean was considered to indicate a high-quality diet.

### Statistical analysis

The participant characteristics are presented based on self-reported diagnosis of cancer (yes/no) and described by age, sex and time since cancer diagnoses (if applicable). To evaluate the relationship between lifestyle factors and being a CSs (yes/no), we dichotomised the lifestyle factors and performed separate logistic regression analyses for each lifestyle factor (Table 1). Multinomial logistic regression was performed for smoking because this was divided into three categories. We input CSs status as the independent variable and the lifestyle factors as the dependent variables. The reference category for the different lifestyle factors in each logistic regression was always the category indicating healthy behaviour. As such, when compared with the cancer-free group, a resulting odds ratio (OR) above 1 indicated that CSs were at increased odds for the unhealthy behaviour, while ORs below 1 indicated that CSs were at increased odds for the healthy behaviour. These analyses were performed with and without adjustment for age and sex. To evaluate if the association between being a CS and having the different lifestyle factors was more prominent in specific subgroups, we stratified the analyses by sex (female or male, also adjusted for age) and by age (< 55 or ≥ 55 years, also adjusted for sex). The age cut-off of 55 years was chosen because this was the mean age of the CSs in our cohort. Finally, we tested if the association between cancer survivorship and lifestyle factors differed between the specific subgroups by entering interaction terms in the logistic regression model. Descriptive statistics and logistic regression analyses were performed in IBM SPSS version 22.0 (IBM Corp., Armonk, NY, USA) and a forest plot was generated in R statistics.

**Table 1:** Lifestyle characteristics of participants in the Lifelines Cohort Study stratified by self-reported cancer history (yes/no; *n* = 115,257)

	Cancer survivors (*n* = 5473 [4.7%])	Those without cancer (*n* = 109,784 [95.3%])	OR
Age (years, mean ± SD)	55.4 ± 12.2	44.37 ± 12.8	
< 55 years old	2599 (47.5%)	86,762 (79%)	
Sex (female)	3647 (66.6%)	64,398 (58.7%)	
Time since diagnosis (years, mean ± SD)	9 ± 8.5	NA	
BMI (kg/m^2^)			
Normal (< 25)	2171 (39.7%)	49,858 (45.4%)	1
Overweight/obese (≥ 25)	3302 (60.3%)	59,926 (54.6%)	1.26 (1.20–1.33)
Physical activity (min/week)			
High activity level (≥ 150)	3396 (62.1%)	65,044 (59.2%)	1
Low activity level (˂ 150)	2077 (37.9%)	44,740 (40.8%)	0.88 (0.84–0.94)
Smoking			
Never smoker	2106 (38.5%)	51,589 (47%)	1
Former smoker	2494 (45.5%)	35,875 (32.7%)	1.70 (1.60–1.80)
Current smoker	873 (16%)	22,320 (20.3)	0.95 (0.88–1.03)
Alcohol intake (g/day)			
No consumer (0 g)	1596 (29.2%)	28,663 (26.1%)	1
Consumer (≥ 1 g)	3877 (70.8%)	81,121 (73.9%)	0.85 (0.81–0.91)
Sedentary behaviour (TV h/day)			
No sedentary (< 2)	1192 (21.8%)	30,673 (27.9%)	1
Sedentary (≥ 2)	4281 (78.2%)	79,111 (72.1%)	1.39 (1.30–1.48)
Diet (LLDS)			
LLDS high-quality diet (≥ 28)	2140 (39.1%)	30,813 (28.1%)	1
LLDS low-quality diet (˂ 28)	3333 (60.9%)	78,971 (71.9%)	0.61 (0.57–0.64)

*SD* standard deviation, *BMI* body mass index, *kg* kilogrammes, *m* meters, *min* minutes, *TV* television, *h* hours, *LLDS* Lifelines diet score

N (%), unless specified otherwise; odds ratios (ORs) refer to univariate analysis with the lifestyle factor as dependent variable and cancer survivorship as independent variable

## Results

A total of 115,257 participants (59% female) were included. Of these, 5473 (4.7%) were CSs and approximately 10 years older than those without a history of cancer (mean age 55.4 ± 12.2 vs 44.4 ± 12.8 years) (Table 1). More females than males reported a history of cancer (66.6% vs 33.4%), and the participants who reported cancer had survived an average of 9 years (± 8.5) since diagnosis.

Compared with the cancer-free group, the CS group were more likely to be overweight or obese (OR = 1.26 [1.20–1.33]), physically active (OR = 0.88 [0.84–0.94]) and former smokers (OR = 1.70 [1.60–1.80]). However, they were also less likely to be alcohol drinkers (OR = 0.85 [0.81–0.91]), more likely to have sedentary behaviours (OR = 1.39 [1.30–1.48]) and more likely to consume healthy diets (OR = 0.61 [0.57–0.64]) (Table 1).

**Fig. 2:**
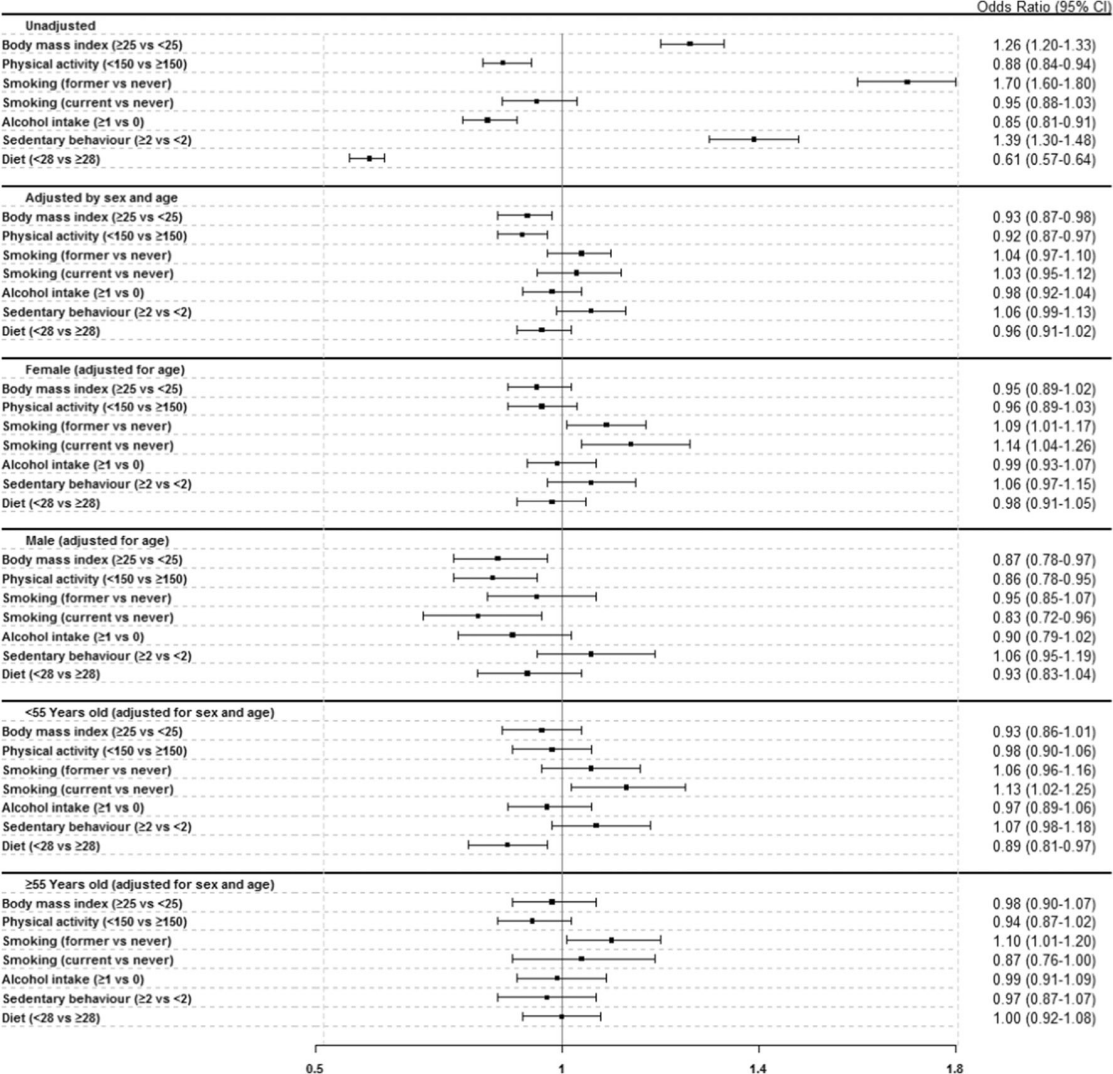
Association between self-reported cancer and six different lifestyle factors*, unadjusted and adjusted analysis, stratified by sex and age

After adjusting the estimates for sex and age, the direction of the association between cancer survivorship and BMI changed, with CSs having lower odds of being overweight or obese than participants with no history of cancer (OR = 0.93 [0.87–0.98]). Additionally, the association between cancer survivorship and physical activity remained statistically significant in this adjusted analysis (OR = 0.92 [0.87–0.97]) (Fig. 2).

Stratified analysis indicated that cancer survivorship in females was positively associated with former smoking (OR = 1.09 [1.01–1.17]) and current smoking (OR = 1.14 [1.04–1.26]). By contrast, compared to males with no history of cancer, those who were CSs had lower odds of being current smokers (OR = 0.83 [0.72–0.96]) and being overweight or obese (OR = 0.87 [0.78–0.97]), while also showing higher levels of physical activity (OR = 0.86 [0.78–0.95]) (see Fig. 2). Interaction analysis showed a significant difference between females and males in the association between cancer survivorship and current smoking (*p =* 0.03).

Age-stratified analysis revealed that cancer survivorship in subjects aged < 55 years was significantly associated with current smoking (OR = 1.13 [1.02–1.25]) and a healthy diet (OR = 0.89 [0.81–0.97]). In subjects aged ≥ 55 years, being a cancer survivor was significantly associated with being a former smoker (OR = 1.10 [1.01–1.20]) (Fig. 2). The interactions between cancer survivorship and age on lifestyle factors were significant for former smoking (*p* = 0.01), current smoking (*p* < 0.001) and diet (*p* = 0.001).

## Discussion

### Summary

In this cross-sectional analysis, we compared the lifestyle data for 109,784 people from the general population with 5473 CSs in the north of the Netherlands. The CSs in our cohort had a mean age of 55 ± 12.2 years, which is consistent with data reported in a recent systematic review of CSs [41], and they had survived an average of 9 years since diagnosis. In general, CSs were more likely to be physically active, less likely to be alcohol drinkers and more likely to have a better diet, yet they were also more likely to have a higher BMI, be former smokers and to have more sedentary behaviour. Notably, only BMI and physical activity retained their statistical significance after adjustment for sex and age, with CSs having slightly more normal BMIs (OR = 0.93 [0.87–0.98]) and higher physical activity levels (OR = 0.92 [0.87–0.97]). Smoking remained a prevalent behaviour in CSs.

After stratification for sex and age, female CSs were more often former and current versus never smokers, whereas male CSs were less often current versus never smokers and more often had higher physical activity than their peers without a history of cancer. In addition, CSs aged ≥ 55 years were more likely to be former smokers than age-matched peers without a history of cancer, and those aged < 55 years were more often former and current smokers. In addition, CSs aged < 55 years had better diets than their peers with no history of cancer.

### Body mass index

In the unadjusted analyses, CSs were more likely to have a BMI ≥ 25 kg/m^2^ than people with no history of cancer, but after adjusting for age and sex, they became more likely to have a normal BMI. This contrasts with other population-based studies in which the association between cancer survivorship and BMI disappeared (adjusted prevalence ratio = 1.00 [0.98–1.01]) [27] or reduced (OR = 1.19 [1.01–1.39]) [28] after adjustment for socioeconomic variables (e.g. age, race, education, income and health care coverage). Our results show a higher prevalence of normal BMI, especially in male CSs, that have not been reported previously.

### Physical activity

CSs were significantly more likely to be physically active than people with no history of cancer in both the unadjusted and adjusted analyses. Stratification also showed that male CSs were more physically active, which has not been reported previously. There is controversy regarding the role of physical activity in the literature. After adjusting for age, education and income, one study found that after there were no significant differences in physical activity levels between CSs and people without a history of cancer [28]. This contrasts with other studies in which it was shown that CSs were more likely to follow physical activity recommendations (OR = 1.09 [1.03–1.16]) [26], were less likely to engage in ≥ 2 h of physical activity per week (adjusted OR = 0.79 [0.67–0.93]) [31] or were more likely to be physically inactive (OR = 1.11 [1.07–1.15]) [27]. Given that these conflicting data on physical activity were collected with different but validated questionnaires that used similar cut-offs, it is possible that the diverse findings can be explained by differences in data collection, time since diagnosis or type of cancer.

### Smoking

Although CSs initially appeared to be more likely to be former smokers, these differences disappeared after adjusting for age and sex. In other studies, it was reported that CSs were more likely to be former smokers after adjustment for socioeconomic variables [26–30, 32, 33]. Interestingly, we also showed that female CSs were more likely to be former or current smokers, similar to data in other studies where the adjusted prevalence ratio was 1.25 (1.18–1.32) [27] and the OR was 2.40 (1.43–4.06) [28]. Furthermore, we showed that male CSs were less likely to be current smokers, which was again consistent with previous findings (adjusted OR = 0.85 [0.79–0.92]) [27]. CSs younger than 55 years old were more often former and current smokers, which compares with the finding of a population-based study in which CSs aged 18–39 were more likely to be current smokers (OR = 1.69 [1.14–2.50]) [28].

### Alcohol consumption

Unadjusted analysis showed that CSs were less likely to drink alcohol than people with no history of cancer, but consistent with other studies, this was not confirmed in the adjusted analysis [28, 29]. There was also no significant difference in the prevalence of alcohol consumption between the CSs and people with no history of cancer after stratification by either sex or age.

### Sedentary behaviour

There was a higher prevalence of sedentary behaviour among CSs in the unadjusted analyses, but the significance of this result was also lost after adjustment by sex and age. We defined sedentary behaviour as the average amount of hours that participants spent watching television, whereas other studies have defined sedentary behaviour as the absence of physical activity. This precludes direct comparison. Given that watching television is an important activity in Western society, we suggest that further research is warranted to assess whether it has a role that is independent of general physical activity levels.

### Diet

It was notable that the CSs were more likely to have healthier diets than their peers with no history of cancer in the unadjusted analyses. However, the differences became nonsignificant in most cases when the models were adjusted for sex and age. Only CSs aged < 55 years remained significantly more likely to have a healthier diet. Some studies have also reported that differences in fruit and vegetable consumption are no different after adjustment for age and sex [27, 28], or that CSs and people with no history of cancer have similar comprehensive diet scores [29]. One study has deviated from this trend, showing that CSs are more likely to eat more fruits and vegetables than people with no history of cancer (OR = 1.41 [1.19–1.66]) [31]. A possible explanation for the different observation in this study is that we included a more comprehensive evaluation of dietary intake and did not limit the assessment to fruit and vegetable consumption alone, resulting in more detailed dietary score calculations. The finding of better diet quality among younger CSs has not been reported in previous studies.

## Strengths and limitations

The main strengths of the present analysis are that we included data from a large population-based cohort that is representative of inhabitants in the north of the Netherlands [35]. The original aim of the Lifelines cohort was to gain insight into the aetiology of healthy ageing in the general population, across generations. Many investigative procedures were performed to collect data on a variety of health and exposure domains, making the cohort suitable for future research, for both healthy subjects and for those with (chronic) diseases such as cancer. This comprehensive design allowed us to answer the current research question. Another strength is that we included six lifestyle factors that were assessed individually by complete case analysis. Food intake was evaluated by a comprehensive diet score and sedentary behaviour was analysed separately from physical activity. To the best of our knowledge, no other study has independently analysed the six lifestyle behaviours included here. Future research should include more detailed and better evaluated behaviours.

As limitations, we must stress that the current data were obtained from a cross-sectional analysis, which precludes any statements of causality. Another limitation is that cancer diagnoses were self-reported and not presented as formal site-specific diagnoses. Given that behavioural lifestyle factors were also self-reported, we cannot exclude the possibility that some participants answered questions according to what they thought was socially desirable. Importantly, we did not adjust our analyses for the potential confounding effect of socioeconomic factors, such as employment, education, and income.

## Conclusion

We conclude that CSs in this population-based cohort have lifestyles that are broadly comparable to those of people with no history of cancer. Nevertheless, adjusted analyses showed that BMI is more likely to be normal, physical activity is more likely to be higher and that smoking remains a prevalent behaviour in CSs. Current smoking also appears to be significantly higher in CSs who are female or aged < 55 years when compared with the general population. Strategies to stop smoking undoubtedly need to target the entire population, but this study shows that special attention may be necessary to motivate and assist females in these two groups.

Although our results are partially in line with those of other studies, some are notably different. A potential explanation for these differences may be that there are cultural differences in lifestyle and health care systems. Another explanation may be the relatively long cancer survivorship of the participants in our cohort, which may have afforded them sufficient opportunity to readapt to the lifestyle of the general population. These represent topics of particular interest to future research. Equally, there is a need to explore causality in the associations of health behaviours in cancer survivorship through longitudinal study.

## Data Availability

The datasets analysed during the current analysis are available from the corresponding author on reasonable request.
